# Positron Emission Tomographic Imaging of Tumor Cell Death Using Zirconium-89-Labeled APOMAB® Following Cisplatin Chemotherapy in Lung and Ovarian Cancer Xenograft Models

**DOI:** 10.1007/s11307-021-01620-1

**Published:** 2021-07-06

**Authors:** Vasilios Liapis, William Tieu, Nicole L. Wittwer, Tessa Gargett, Andreas Evdokiou, Prab Takhar, Stacey E. Rudd, Paul S. Donnelly, Michael P. Brown, Alexander H. Staudacher

**Affiliations:** 1 Translational Oncology Laboratory, Centre for Cancer Biology, SA Pathology and University of South Australia, Level 9 University of South Australia Health Innovation Building, North Terrace, Adelaide, 5000 Australia; 2 School of Medicine, University of Adelaide, Adelaide, SA 5000 Australia; 3 Molecular Imaging and Therapy Research Unit (MITRU), South Australian Health and Medical Research Institute (SAHMRI), Adelaide, Australia; 4 Discipline of Surgery, Breast Cancer Research Unit, Basil Hetzel Institute and Centre for Personalised Cancer Medicine, University of Adelaide, Woodville, SA 5011 Australia; 5 School of Chemistry and Bio21 Molecular Science and Biotechnology Institute, University of Melbourne, Melbourne, Victoria 3010 Australia; 6 Cancer Clinical Trials Unit, Royal Adelaide Hospital, Adelaide, SA 5000 Australia

**Keywords:** Zirconium^89^, chDAB4, Chemotherapy, Ovarian cancer, Lung cancer

## Abstract

**Purpose:**

Early detection of tumor treatment responses represents an unmet clinical need with no approved noninvasive methods. DAB4, or its chimeric derivative, chDAB4 (APOMAB®) is an antibody that targets the Lupus associated antigen (La/SSB). La/SSB is over-expressed in malignancy and selectively targeted by chDAB4 in cancer cells dying from DNA-damaging treatment. Therefore, chDAB4 is a unique diagnostic tool that detects dead cancer cells and thus could distinguish between treatment responsive and nonresponsive patients.

**Procedures:**

In clinically relevant tumor models, mice bearing subcutaneous xenografts of human ovarian or lung cancer cell lines or intraperitoneal ovarian cancer xenografts were untreated or given chemotherapy followed 24h later by chDAB4 radiolabeled with [^89^Zr]Zr^IV^. Tumor responses were monitored using bioluminescence imaging and caliper measurements. [^89^Zr]Zr-chDAB4 uptake in tumor and normal tissues was measured using an Albira SI Positron-Emission Tomography (PET) imager and its biodistribution was measured using a Hidex gamma-counter.

**Results:**

Tumor uptake of [^89^Zr]Zr-chDAB4 was detected in untreated mice, and uptake significantly increased in both human lung and ovarian tumors after chemotherapy, but not in normal tissues.

**Conclusion:**

Given that tumors, rather than normal tissues, were targeted after chemotherapy, these results support the clinical development of chDAB4 as a radiodiagnostic imaging agent and as a potential predictive marker of treatment response.

**Supplementary Information:**

The online version contains supplementary material available at 10.1007/s11307-021-01620-1.

## Introduction

The care and treatment of cancer patients places an intense demand on healthcare systems globally. Standard treatment regimens for locally advanced or metastatic cancers usually employ radiotherapy, cytotoxic chemotherapy or both. Often, however, the treatment is not sufficiently effective and may represent a significant personal and socioeconomic burden for patients if it is both toxic and ineffective. Therefore, a robust method for early or interim determination of tumor responses may allow patients to continue effective therapy, discontinue ineffective therapy and also aid in go/no-go decision making in anticancer drug development.

Current methods for determining tumor responses are based on measuring changes in tumor dimensions using computed tomography (CT), magnetic resonance imaging (MRI), and ultrasound according to Response Evaluation Criteria in Solid Tumors (RECIST) [1] or measuring FDG avidity on ^18^F-FDG positron emission tomography (PET)/(PET/CT) scans using PET Response Criteria in Solid Tumors (PERCIST) or tumor-specific criteria [2, 3]. Although there is no established role for FDG-PET in chemotherapy response monitoring of either lung or ovarian cancers [4, 5], FDG-PET and SPECT studies in nonsmall cell lung carcinoma (NSCLC) patients after a first cycle of platinum-based chemotherapy have been informative. For example, tumor responders, who were defined as having a decrease of at least 10 % in standardized uptake value (SUV) of tumor FDG uptake, had the best survival prospects [5, 6]. Nevertheless, the sensitivity and specificity of FDG-PET/CT for therapy response monitoring may be limited by such tumor variables as expression of glucose transporters [7], or tissue inflammation or infection, both of which may also be FDG avid [8]. Therefore, there has been great interest in the field of molecular imaging to develop selective, robust, and clinically applicable *in vivo* imaging markers of therapy-induced tumor cell death [9–11].

For example, in NSCLC patients who received ^99m^Tc-labeled annexin V as a marker of cell death, early chemotherapy-induced tumor-specific annexin V uptake on SPECT significantly correlated with later RECIST-defined tumor responses although some patients with RECIST-defined stable disease also had tumor annexin V uptake [12]. However, increased tumor uptake of annexin V appeared to depend on the exact time after the start of chemotherapy and the type and stage of the cancer treated, and high accumulation of annexin V in the kidneys was also evident [10, 13].

Further among the cited *in vivo* imaging markers of therapy-induced tumor cell death that may possess the requisite qualities is DAB4, which originated as a mouse monoclonal antibody (mAb) [14, 15] and which has been reformatted as a chimeric mAb (chDAB4; APOMAB®) [16, 17]. In either format, the DAB4 and chDAB4 mAbs target the ubiquitously and abundantly expressed ribonucleoprotein, Lupus-associated (La)/Sjögren Syndrome-B (SSB) antigen, which is essential for life [18, 19]. As a multifunctional RNA binding protein, La/SSB protects nascent RNAs from exonucleases enabling their maturation [20], and also has roles promoting both microRNA biogenesis [21] and translation of select mRNAs as an IRES transacting factor (ITAF) [22]. Consequently, as we and others have shown, La/SSB is overexpressed in many different cancers [23–27] and contributes to the malignant process [28].

La/SSB only becomes accessible for antibody binding in cells that have lost membrane integrity, particularly in apoptotic and necrotic cancer cells, making DAB4 a dead tumor cell–targeting mAb, particularly after DNA-damaging anticancer treatment [14, 15]. The La/SSB protein is highly conserved between mice and humans and DAB4 binds to both mouse and human forms of La/SSB. We have shown both *in vitro* and *in vivo* that DAB4 binds with high specificity to dead tumor cells [23, 29]*.* We drew on this tumor-targeting property to radiolabel DAB4 with ^111^In for tumor imaging [29, 30] and with ^90^Y [15], ^177^Lu [27] and ^227^Th [31] for antitumor therapy by exploiting the phenomenon of β- and α-radiation crossfire [32].

In preparation for a newly commenced clinical radiodiagnostic imaging trial, the variable region sequences of murine DAB4 were genetically fused to the constant region sequences of human IgG1 to generate chimeric DAB4 (chDAB4) [17]. We radiolabeled chDAB4 with the positron-emitting radionuclide Zirconium-89 ([^89^Zr]Zr^IV^) so that the radioimmunoconjugate could be used for noninvasive PET imaging. The physical half-life of [^89^Zr]Zr^IV^ of 3.3 days is similar to the biological half-life of mAbs, so the radionuclide is well suited for radiolabeling mAbs, making it possible to obtain PET images with desirable contrast between tumor uptake and normal tissue background. Consequently, [^89^Zr]Zr^IV^ has been used extensively for radiolabeling antibodies for clinical immunoPET studies [33, 34].

In this study, the ability of [^89^Zr]Zr-chDAB4 to selectively bind to dead tumor cells in human xenografts of lung and ovarian cancer after chemotherapy was examined using PET imaging. This preclinical validation study supports the use of chDAB4 as a diagnostic tool for detecting tumor responses to first line platinum-based chemotherapy in lung and ovarian cancer patients.

## Materials and Methods

### Cell Culture and Antibodies

The A2780 ovarian cancer cell line was purchased from ATCC (USA) and the H460 large cell lung carcinoma cell line was a gift from Associate Professor Carleen Cullinane (Peter MacCallum Cancer Centre, Australia) and both were authenticated by short tandem repeat testing using the AmpFISTR Identifier Kit (ThermoFisher Scientific) by SA Pathology (Adelaide, South Australia). Raji cells were purchased from CellBank (Australia) and Jurkat cells were kindly provided by Professor Andrew Zannettino (Myeloma Research Laboratory, University of Adelaide, Australia). All cell lines were cultured in RPMI-1640 which contained penicillin and streptomycin (Sigma-Aldrich) and 10 % foetal calf serum (FCS) (Bovogen Biologicals, Victoria, Australia).

Cells were negative for mycoplasma using MycoAlert® Mycoplasma Detection Kit (Lonza, Basel, Switzerland). The generation of luciferase tagged H460 and A2780 cells was performed as described previously [35–37]. Chimeric DAB4 (chDAB4), resulting from the genetic fusion of the variable region sequences of murine DAB4 to the constant region sequences of human IgG1 (huIgG1) was created at CSIRO Manufacturing and produced using the CHO-XL99 system at the National Biologics Facility, Australian Institute for Bioengineering and Nanotechnology, University of Queensland (Queensland, Australia) [16, 17]. The CH2 domain of chDAB4’s huIgG1 Fc harbors the K322A mutation, which can abrogate complement-dependent cytotoxicity (CDC) and attenuate antibody-dependent cytotoxicity (ADCC) [38].

### Flow Cytometry Analysis of chDAB4 Binding to H460 and A2780 Cells

A2780 and H460 cells were treated with increasing concentrations of cisplatin (Hospira, Australia) for 48 h, collected, washed twice by centrifugation at 900*g* for 5 min with FACS buffer (2.5 % FCS, 0.04 % sodium azide in PBS) and incubated with 5 μg/mL chDAB4 or human IgG (Intragram P; as control) for 30 min. Cells were washed and incubated with 2 μg/mL goat antihuman IgG Alexa Fluor® 647 (ThermoFisher Scientific, USA) for 20 min, washed further, incubated for 10 min with 0.5 μg/mL Propidium Iodide (PI) (Sigma-Aldrich) and analyzed by flow cytometry using a BD Accuri flow cytometer (BD Biosciences, Franklin Lakes, NJ, USA). Gating was set at 2 % positive events in the isotype control sample. Specific binding was calculated as the difference in Mean Fluorescence Intensity (MFI) between chDAB4 and the isotype control antibody and expressed as the net MFI, which was calculated from triplicate samples.

### Chelator Conjugation and Radiolabeling of chDAB4 With [^89^Zr]Zr^IV^

[^89^Zr]Zr^IV^ oxalate was produced by SAHMRI via proton irradiation of a ^89^Y target on a PETtrace 880 cyclotron (GE Healthcare) and purified on an Alceo solid target processing system (Comecer, Italy) as described previously [39]. In this preclinical study, the chDAB4 mAb was conjugated to the bifunctional chelator *p*-isothiocyanatobenzyl-deferoxamine (DFO-*p*Phe-NCS) (Macrocyclics, Texas, USA) as described [40] and radiolabeled with [^89^Zr]Zr^IV^. For simplicity of expression, the [^89^Zr]Zr^IV^-radiolabeled DFO-*p*Phe-NCS conjugate of chDAB4 is henceforth referred to as [^89^Zr]Zr-chDAB4. On the other hand, based on recent preclinical data comparing the DFO-*p*Phe-NCS conjugate of chDAB4 with a newly developed DFO-Sq (H_3_DFOSqOEt) conjugate of chDAB4 [41], a [^89^Zr]Zr^IV^-radiolabeled DFO-Sq conjugate of chDAB4 was employed in the ongoing phase 1 clinical trial (ANZCTR No. 12620000622909) radiolabeled with [^89^Zr]Zr^IV^ as described previously [40]. The radiolabeled antibodies were washed and concentrated using 50 kDa MWCO centrifuge filters and resuspended in sterile PBS for injection. Instant thin layer chromatography (ITLC) with 25 mM citrate buffer (pH 5.5) as a mobile phase was used to measure the amount of free [^89^Zr]Zr^IV^ in the preparation, which was <1 % of total activity (R_f_ <0.2 [bound [^89^Zr]Zr^IV^] *vs* R_f_ >0.7 [free [^89^Zr]Zr^IV^)]).

### Cell Binding Assays

A modified version of the Lindmo binding assay [42] was used to determine the immunoreactive fraction (IRF) of the radiolabeled chDAB4. H460 human lung cancer cells were fixed and permeabilized as previously described to expose the La/SSB protein [14, 15], resuspended in PBS with 1 % FCS and serially diluted from 5 × 10^7^ to 3.9 × 10^6^ cells in 0.5mL in duplicate. 500 ng/mL of radiolabeled antibody in 0.5 mL PBS with 1 % FCS was added to the cell suspensions and incubated at 4 °C overnight. The cells were pelleted by centrifugation at 900*g* and 0.5 mL of the supernatant from each sample was placed in a separate tube. The radioactivity in the cell pellets and the supernatant was measured using an automatic Gamma Counter (Hidex, Finland). The inverse cell concentration (mL/cells in sample) was plotted against the total activity (activity in supernatant + activity in pellet)/specific binding (total bound) and the linear regression was extrapolated to calculate interception of the y-axis where x = 0 and expressed as a percentage (IRF = 100 % × 1/y[x=0]). The binding affinity of conjugated and radiolabeled chDAB4 was compared to the intact, unlabeled chDAB4 using a La/SSB-specific ELISA as described previously [16].

### *In Vitro* Assays for Complement Dependent Cytotoxicity (CDC), Antibody Dependent Cellular Cytotoxicity (ADCC) and Cytokine Release

In the CDC assay, CD20^+^ Raji cells or CD20^-^ Jurkat cells were harvested, washed and added to each well of a 96-well U-bottom plate at 10^5^ cells/well in RPMI-1640 with 10 % rabbit serum as the source of complement. The CD20-specific chimeric mAb, rituximab, was used as a positive control, and medium alone and the EGFR-specific chimeric mAb, cetuximab, were used as negative controls. The mAbs were added at a concentration of 15, 1.5, 0.15 or 0.015 μg/mL in RPMI-1640 with 10 % rabbit serum and incubated for 2 h at 37 °C. Thiazolyl Blue Tetrazolium Bromide at a final concentration of 0.4 mg/mL was added per well and the plate incubated for 1 h at 37 °C with 5 % CO_2_. The plate was centrifuged at 300*g* for 3 min, the supernatant was removed, and the resulting crystals resuspended in 150 μL isopropanol. Absorbance was measured at 570 nm using a FLUOStar Omega plate reader.

ADCC was examined using the Promega ADCC Reporter Bioassay kit. The assay uses genetically modified Jurkat effector cells, which stably express the high-affinity V158F receptor variant of FcγRIIIa together with a reporter gene construct comprising a nuclear factor of activated T-cells (NFAT) response element driving firefly luciferase expression. Binding to FcγRIIIa via the mAb Fc domain results in detectable luciferase expression and induction of ADCC. CD20^+^ Raji cells were used as the target cells. Permeabilized H460 cells, which are strongly bound by chDAB4, were also used as target cells. In a 96-well plate, chDAB4 or rituximab were added at 3 μg/mL in triplicate. The antibodies were serially diluted 1:2.5 to achieve final concentrations of 0, 0.002, 0.005, 0.012, 0.03, 0.08, 0.19, 0.48, 1.2, or 3 μg/mL. The target cells were added to each well followed by effector cells and incubated for 6 h at 37 °C with 5 % CO_2_. Plates were equilibrated to room temperature for 15 min before the Bio-Glo Luciferase Assay Reagent was added, incubated for 45 min and luminescence measured using a FLUOstar Omega plate reader.

For the cytokine release assay (CRA), fresh blood was taken from five volunteer donors. Peripheral blood mononuclear cells (PBMCs) were isolated using density gradient centrifugation with Lymphoprep (Alere Technologies) as per manufacturer’s instructions and seeded at 10^5^ cells/well in a 96-well U-bottom plate. In duplicate, cells were treated with increasing doses of chDAB4 or 15 μg/mL anti-CD3 antibody (clone OKT3; prepared in-house) as a positive control for 24 h at 37°C with 5 % CO_2_. The following day, cells were pelleted and 100 μL of supernatant per well was analyzed for cytokine levels using the LEGENDplex Human Inflammation Panel 1 (Biolegend) following the manufacturer’s instructions.

### Animal Experiments

The SAHMRI Animal Ethics Committee, (Adelaide, Australia) approved all animal experiments, which were conducted following institutional ethical guidelines. Six to ten-week-old female NOD *scid* gamma-null (NSG) mice were inoculated subcutaneously in the right flank with 5×10^6^ A2780 or H460 tumor cells in a 1:1 ratio of Matrigel/PBS. For the intraperitoneal ovarian cancer model, 5×10^6^ A2780 cells in 100 μL PBS were injected into the intraperitoneal cavity.

The IVIS® Spectrum Imaging system (PerkinElmer, Massachusetts, USA) was used for noninvasive monitoring of tumor growth of the luciferase-expressing cell lines. Mice were given intraperitoneal injections (i.p.i.) with 100 μL of D-Luciferin solution at 150 mg/kg (Pierce Biotechnology, IL, USA) and then gas-anesthetized with isoflurane (Veterinary Companies of Australia, NSW, Australia). Images were acquired for 1–30 sec (representative images are shown at 1 sec, 20 min after D-Luciferin by i.p.i.) and the photon emission transmitted from mice was captured and quantified as photons/sec/cm^2^ using Living Image Software (version 4.7.2; Perkin Elmer, Massachusetts, USA). Tumor growth was also measured using electronic calipers with tumor volume determined using the calculation (a^2^ x b)/2, where a is the shortest diameter and b is the longest diameter of the tumor.

Mice were randomly allocated to treatment groups when the subcutaneous tumors reached approximately 50 mm^3^, or 7 days after intraperitoneal injection of A2780 cells. Mice were untreated or treated by i.p.i. with 4 mg/kg cisplatin on Day 0. The day following chemotherapy (Day 1), mice bearing subcutaneous tumors were given 6 MBq/50 μg [^89^Zr]Zr-chDAB4 by intravenous injection (i.v.i.) while mice bearing ascitic A2780 tumors were given 6 MBq/75 μg of [^89^Zr]Zr-chDAB4 by i.v.i.. As a blocking control, mice bearing H460 tumors were given 250 μg of unlabeled chDAB4 i.v.i. 1 h before the [^89^Zr]Zr-chDAB4 injection. Mice were monitored daily, and tumor volume measured at least three times per week via bioluminescence imaging and caliper measurement. At the end of the experiment, mice were humanely killed by cervical dislocation, and organs were removed, weighed and radiation counts were measured with background and decay corrected using a Hidex gamma-counter for accumulation of [^89^Zr]Zr^IV^.

### Animal PET and MRI Scanning

Mice were anesthetized with 2 % isoflurane and scanned for 10 min using the Albira Si PET-SPECT small animal scanner (Bruker Biospin GmbH, Valencia, Spain), with a submillimetric resolution of 0.7 mm. Using the PMOD imaging suite (PMOD technologies, Switzerland), regions of interest were manually drawn around the tumor from the Maximum Intensity Projection (*MIP*) sections assisted by the automatic 3D setting in PMOD software. Tumor margins and thresholds were automatically detected by the software, which includes a standardized cut off determined by PMOD. These tumor margins and cut off thresholds were used to analyze the radiotracer accumulation within the tumor. At the end of the study, 2 mice with intraperitoneal A2780 tumors underwent MRI imaging using the T2 weighted RARE spin echo sequence on the Bruker Icon 1T benchtop MRI system. The ITK Snap software was used to segment the scans [43].

### PET/CT Imaging of Patients

Based on the demonstration of superior preclinical PET imaging qualities of the [^89^Zr]Zr-labeled DFO-Sq conjugate of chDAB4 compared to its DFO-*p*Phe-NCS conjugate [41], we adopted the DFO-Sq conjugate of chDAB4 in a recently commenced phase I clinical PET imaging trial of [^89^Zr]Zr-chDAB4. This trial was approved by the Central Adelaide Local Health Network Human Research Ethics Committee and is registered as No. 12620000622909 29/05/2020 with the Australian and New Zealand Clinical Trials Registry (ANZCTR) https://www.anzctr.org.au/Trial/Registration/TrialReview.aspx?id=379693. Patients with advanced lung cancer received a single intravenous injection of a 1.2 mg mass dose of the DFO-Sq conjugate of chDAB4, which had been labeled with 37 MBq [^89^Zr]Zr^IV^. Patients underwent serial PET/CT scans on a Siemens Biograph mCT Flow after having a standard FDG-PET/CT scan on the same scanner within the previous 7–14 days. All patients provided and signed informed consent to participate in the study.

### Statistical Analysis

Statistical analyses were performed using GraphPad Prism (v7.0) software. Comparison of groups was performed by two-way *t*-test or intergroup comparisons made by two-way Analysis of Variance (ANOVA). Data are shown as mean ± Standard Error of the Mean (SEM). Statistical significance was reached when *p* < 0.05, with * representing *p* < 0.05, ** *p* < 0.01, *** *p* < 0.001, and **** *p* < 0.0001. All methods and studies were performed in accordance with the guidelines and regulations stated by the University of South Australia, the University of Adelaide, SAHMRI and the Royal Adelaide Hospital.

## Results

### chDAB4 Binds to Cisplatin-Treated Dead H460 Lung Cancer Cells and A2780 Ovarian Cancer Cells *In Vitro*

**Fig. 1.**
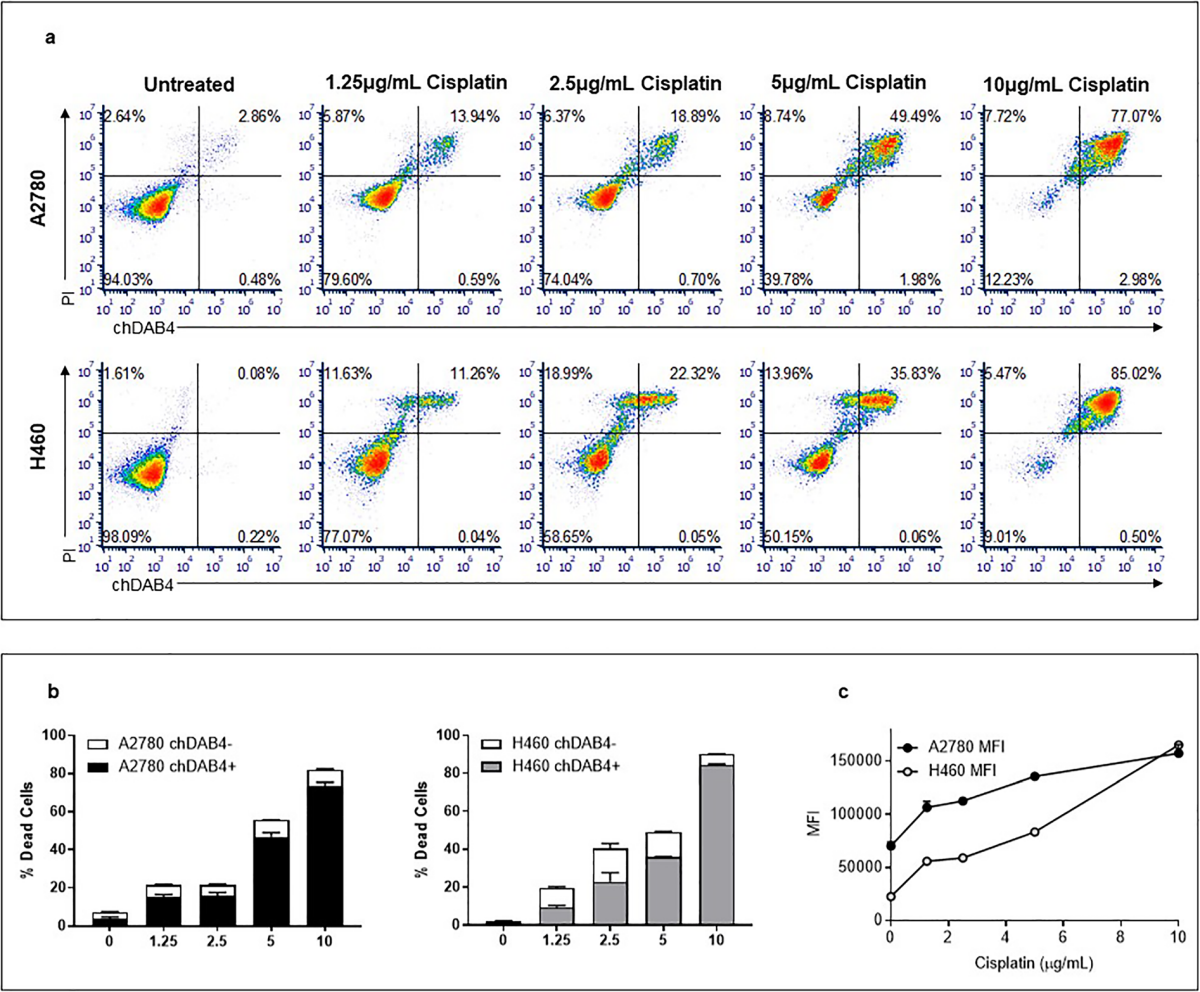
chDAB4 binds to human A2780 ovarian cancer and H460 lung cancer cells after cisplatin treatment in vitro. **a.** A2780 and H460 cell lines were treated with increasing doses of cisplatin for 48 h and cell death (PI^+^) and chDAB4 binding analyzed by flow cytometry. **b.** Increasing the concentration of cisplatin resulted in significantly increased cell death and chDAB4 binding for both A2780 and H460 cells (*p*<0.0001 for 10 μg/mL cisplatin compared to no cisplatin). **c.** After subtracting the mean fluorescence intensity (MFI) of binding of Intragram P used as the isotype control, the MFI of chDAB4 bound to PI^+^ H460 or A2780 cells increased with cisplatin treatment. All data points are means ± SEM.

The A2780 and H460 cell lines were untreated or treated for 48 h with increasing doses of cisplatin and the binding of chDAB4, or the human IgG control, to dead (PI^+^) cells was assessed. For both cell lines, cisplatin treatment increased cell death in a dose-dependent manner (Fig. 1a). Cisplatin also increased the percentages of chDAB4-bound dead tumor cells and, at the highest dose of 10 μg/mL of cisplatin, >85 % of H460 cells and 80 % of A2780 cells were PI^+^ and bound by chDAB4 (Fig. 1b). This dose-dependent increase in proportions of chDAB4-bound dead tumor cells was matched by proportionate increases in the per cell binding of chDAB4, which was measured as increasing mean fluorescence intensity (MFI) of dead tumor cell-bound chDAB4 with increasing cisplatin dose (Fig. 1c). These data confirmed that chDAB4 bound only to treatment-induced dead tumor cells for these two cancer lines.

### Validation of Conjugation and Radiolabeling of Immunoreactive chDAB4

**Fig. 2.**
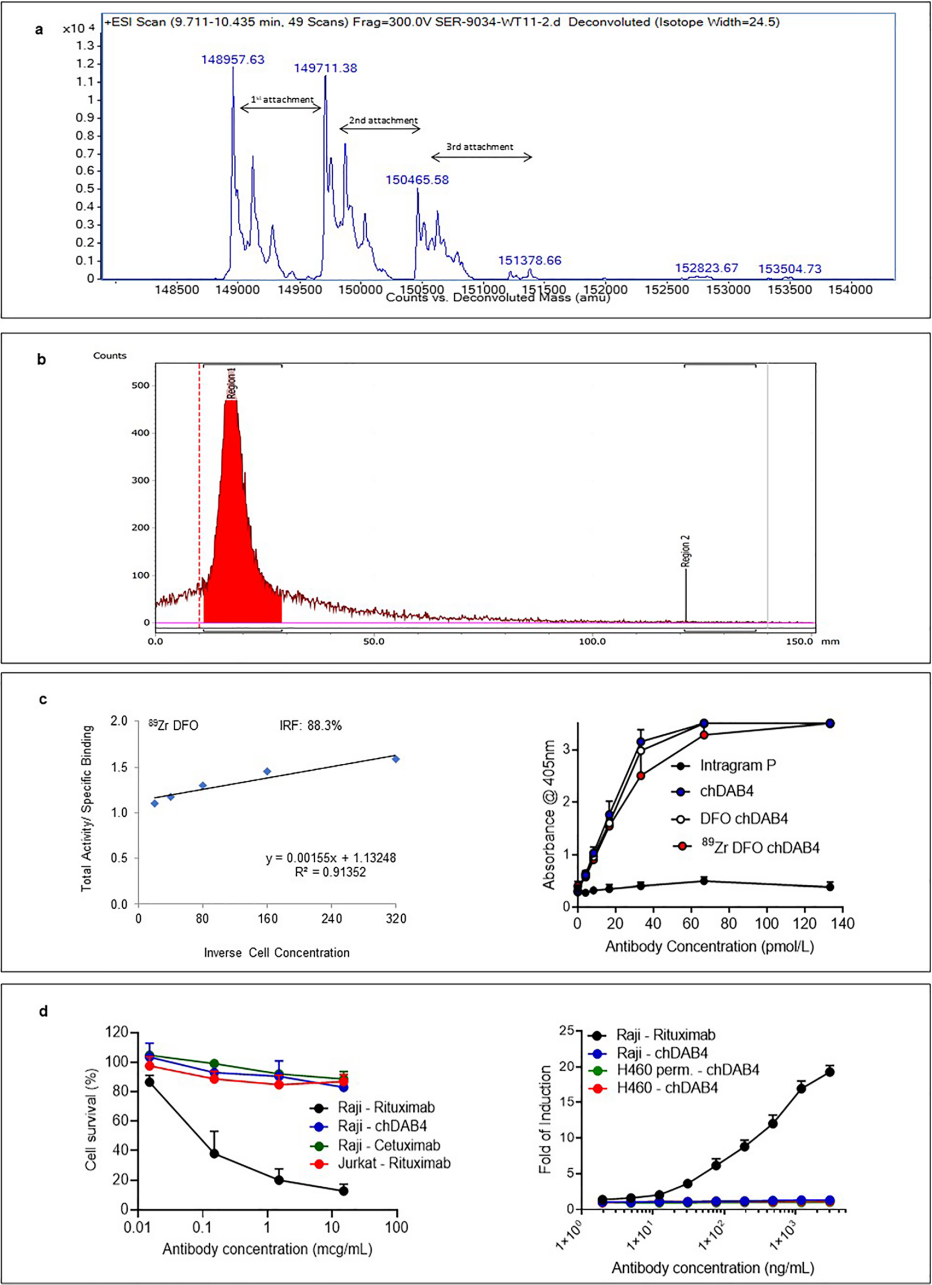
Conjugation of chDAB4, immunoreactivity of unconjugated and conjugated chDAB4 and the lack of immune effector functions of conjugated chDAB4. ***a*.** Mass spectrometry shows 0–3 attachments of DFO with an average of 0.8 chelator molecules per antibody. **b.** ITLC results show <1 % free [^89^Zr]Zr^IV^ in the preparation. **c.** Left; Results of a representative Lindmo assay, which shows an immunoreactive fraction (IRF) value of 88.3 % for the [^89^Zr]Zr-labeled DFO-conjugate of chDAB4. **c.** Right; Binding to the La peptide epitope by unconjugated chDAB4 and by [^89^Zr]Zr-labeled or unlabeled DFO-conjugates of chDAB4 as measured by ELISA (*n* = 3). **d.** Left; Mean % cell survival of CD20^+^ Raji cells or CD20^¯^ Jurkat cells in the CDC assay in response to varying concentrations of chimeric monoclonal antibodies (mAbs) (*n* = 3). **d.** Right; Mean fold induction of reporter gene expressed by effector Jurkat cells in the ADCC assay in response to the chimeric mAbs, chDAB4 and CD20-specific rituximab. Target cells are CD20^+^ Raji or CD20^¯^ H460 cells. The H460 cells were either viable or permeabilized (perm.) to expose the La/SSB protein (*n* = 3).

After DFO conjugation to the chDAB4 mAb, analysis by electrospray ionization mass spectrometry revealed the conjugate had between 0 and 3 chelators attached per mAb with an average of 0.8 H_3_DFO per antibody (Fig. 2a). Radiolabeling of chDAB4 with [^89^Zr]Zr^IV^, resulted in specific activities ranging from 115 to 170 MBq/mg and <1 % free [^89^Zr]Zr^IV^ in the preparation by ITLC (Fig. 2b), with the immunoreactive fraction being 88.3 % as determined by the Lindmo assay (Fig. 2c). The binding of unconjugated, conjugated or radiolabeled versions of chDAB4 to the La/SSB peptide epitope measured by ELISA were similar (Fig. 2c), with the dissociation constant (Kd) values for unconjugated chDAB4, DFO-conjugated chDAB4 and the radioimmunoconjugate [^89^Zr]Zr-DFO-chDAB4 being 17.8 ± 2.8, 19.7 ± 2.6, and 23.1 ± 3.9 pmol/L (± SEM) respectively (Fig. 2c). These data show that binding affinities were minimally altered by the processes of DFO conjugation or radiolabeling.

### Characterization of the Immune Effector Properties of chDAB4

We sought to mitigate the risk of retaining immune effector functions of CDC and ADCC inherent in a version of chimeric DAB4 with a wildtype Fc region, which might exert untoward bystander pro-inflammatory effects, by introducing the K322A mutation [38] in the Fc region of chDAB4. Then, we investigated the immunologic properties of this unconjugated and K322A mutant version of chDAB4 using *in vitro* assays for CDC, ADCC and cytokine release. Although the anti-CD20 targeting mAb rituximab initiated the complement-mediated lysis of CD20^+^ Raji target cells but not CD20^-^ Jurkat cells, neither of the other chimeric mAbs, chDAB4, and cetuximab, both of which contain a huIgG1 Fc domain, resulted in cytotoxicity of the Raji cells (Fig. 2d). Next, we used a reporter assay to measure the intracellular signaling that would be induced in cells mediating ADCC after exposure to antibody-bound target cells. In contrast to the induction of the reporter signal after exposure to rituximab-bound Raji cells, there was no signal induction either using as targets, the viable Raji or H460 cells that show minimal or no binding by chDAB4, or the permeabilized H460 cells that show high binding by chDAB4 (Fig. 2d). Results of the *in vitro* cytokine release assay (Supplementary Figure 1) show a relative lack of reactivity of chDAB4 among PBMCs of 5 normal subjects and, in particular, no chDAB4-dose-dependent elevations in inflammatory cytokine levels were observed. One subject (1), who was found to have had a concurrent systemic viral infection, had an increase in IL-1β, IL-6, IL-10, and TNF-α following chDAB4 incubation which appeared to be inversely proportion to chDAB4 concentration used. Another subject (2) had elevations of IL-6 and TNF-α that were independent of chDAB4 concentration.

### [^89^Zr]Zr-chDAB4 Biodistribution and Tumor Uptake in Untreated and Cisplatin Treated Mice Bearing Subcutaneous Xenografts of the Human H460 Lung Cancer Cell Line

**Fig. 3.**
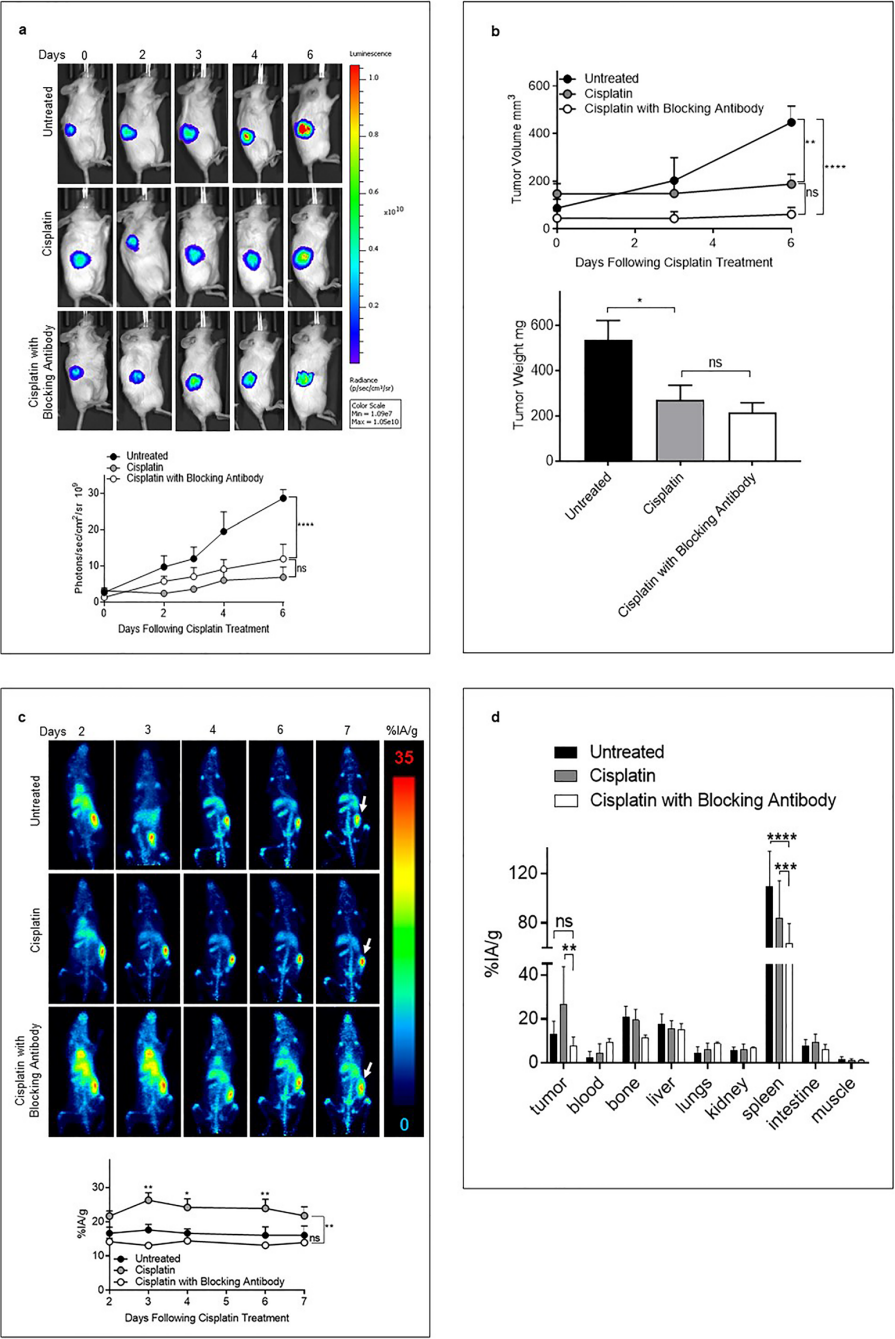
Biodistribution of [^89^Zr]Zr-chDAB4 in mice bearing subcutaneous xenografts of H460 human lung cancer. **a**. Representative longitudinal whole-body bioluminescence images of a single mouse from each group (*n* = 5) are shown. Color scales indicates relative luminescence (photon flux). The line graph shows relative tumor growth measured as the average tumor bioluminescence signal over time (expressed as mean photon counts per second per cm^2^). **b**. The tumor volume calculated from caliper dimensions on Days 0, 3, and 6 (top panel) and the weight of tumors removed at end of study on Day 7 (bottom panel). **c.** Representative spatial Maximum Intensity Projections of whole-body PET images of a single mouse from each group are shown. Arrows at Day 7 time point indicate tumors in right flank of each mouse. Tumor uptake of [^89^Zr]Zr-chDAB4 during the experiment was quantified and expressed as the percentage injected activity per gram (%IA/g) using PMOD® software (bottom panel). **d**. Organs were removed on Day 7 and the biodistribution of [^89^Zr]Zr-chDAB4 was measured using a HIDEX counter (right-hand panel). All data points are means ± SEM and P values were determined by two-way ANOVA.

Mice bearing subcutaneous xenograft model of H460 lung cancer were either untreated or treated with cisplatin (Day 1) followed the next day (Day 2) by injection of [^89^Zr]Zr-chDAB4. To demonstrate the antigen specificity of the tumor uptake of [^89^Zr]Zr-chDAB4, a group of mice received a five-fold molar excess (250 μg) of unlabeled, cold chDAB4 1 h before the [^89^Zr]Zr-chDAB4 injection to act as a blocking control. Cisplatin treatment significantly reduced tumor size as measured by bioluminescence imaging (Fig. 3a), caliper measurements, and tumor weights at the end of study (Fig. 3b). As shown in Fig. 3c, data obtained over the 6-day PET imaging period demonstrated the greatest tumor uptake on Day 3 of [^89^Zr]Zr-chDAB4 as percentage injected activity per gram (%IA/g ± SEM) in cisplatin-treated mice (26.9 ± 2.2) compared to cisplatin-treated mice given prior blocking antibody (13.0 ± 0.7), or untreated mice (17.6 ± 1.6). Moreover, it is also apparent from Fig. 3c that tumor uptake of [^89^Zr]Zr-chDAB4 after cisplatin treatment significantly increased on Days 3 and 4 before tumor growth delay became apparent (Fig. 3b), and all while PET images during the week post-injection showed diminishing background uptake (Fig. 3c). *In vivo* antigen blockade [44] appreciably reduced tumor accumulation of [^89^Zr]Zr-chDAB4 and prolonged blood-pool radioactivity (Fig. 3c) thereby confirming that the tumor targeting by [^89^Zr]Zr-chDAB4 was specific. The physical biodistribution data obtained at the completion of the study (Fig. 3d) showed significantly greater mean tumor uptake of [^89^Zr]Zr-chDAB4 in cisplatin-treated mice measured as %IA/g ± SEM of 26.7 ± 7.6 compared to 7.7 ± 1.8 for cisplatin-treated mice given prior cold chDAB4 mAb as a blocking control, but not significantly different compared to untreated mice with %IA/g ± SEM of 13.1 ± 2.6. Consistent with this result, we found that the mean %IA/g ± SEM of 9.4 ± 0.7 in blood of cisplatin-treated mice after prior blocking antibody was higher than in cisplatin-treated mice (4.3 ± 2.0) and in untreated mice (2.1 ± 1.3).

Conversely, prior blocking antibody led to a lower mean bone uptake ± SEM of 11.5 ± 0.5 %IA/g compared to %IA/g values of 20.7 ± 2.3 and 19.5 ± 2.1 in cisplatin-treated mice and untreated mice, respectively. Interestingly, in cisplatin-treated mice pre-administered blocking antibody, mean splenic uptake ± SEM as %IA/g of radiolabeled antibody was 63.2 ± 7.3 and significantly reduced compared to 83.7 ± 13.6 in mice treated with cisplatin alone; and 109.3 ± 12.9 in untreated mice.

### [^89^Zr]Zr-chDAB4 Biodistribution in Untreated and Cisplatin-Treated Mice Bearing Subcutaneous and Ascitic Xenografts of the Human A2780 Ovarian Cancer Cell Line

**Fig. 4.**
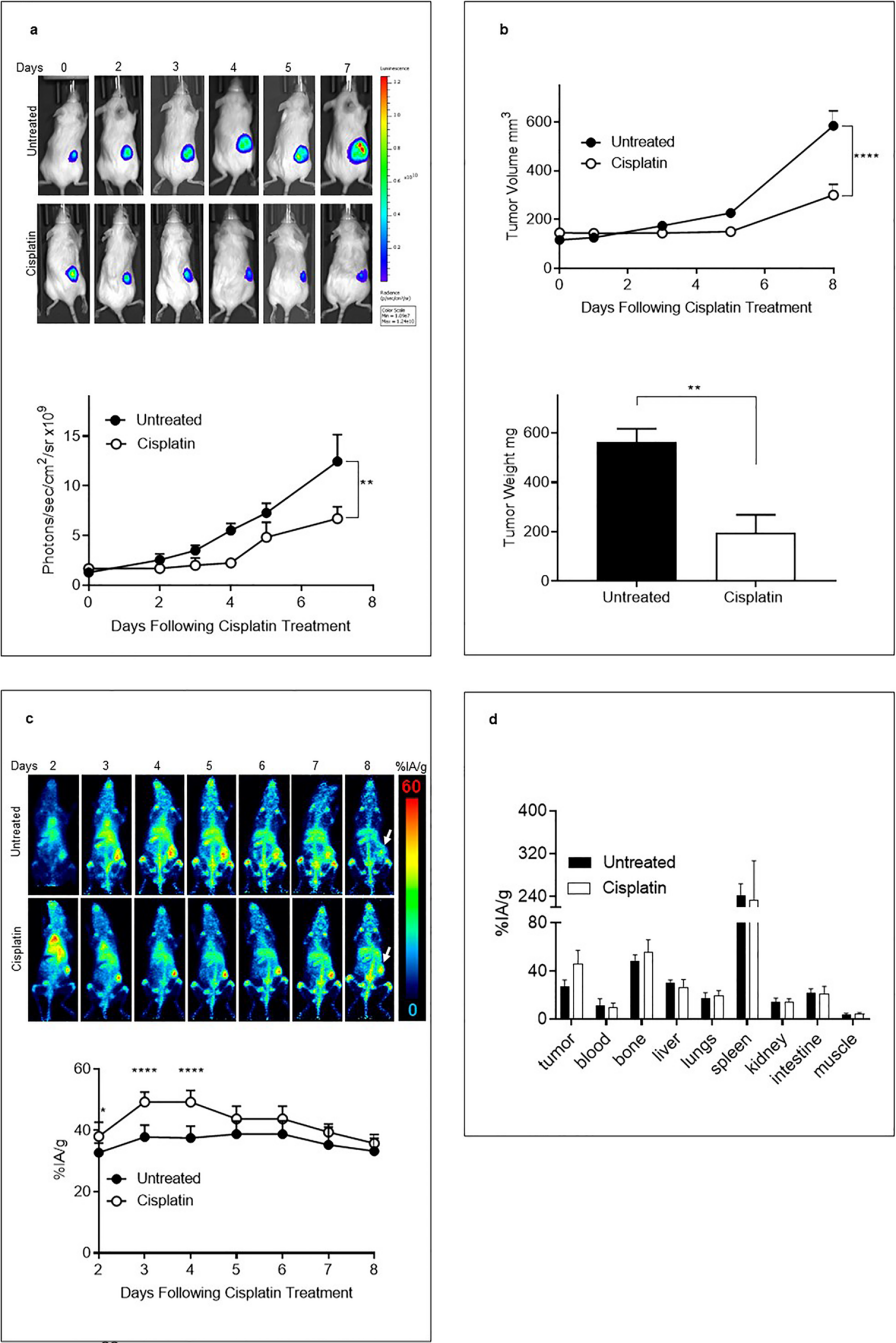
Biodistribution of [^89^Zr]Zr-chDAB4 in mice bearing subcutaneous xenografts of A2780 human ovarian cancer. **a.** Representative longitudinal whole-body bioluminescence images of a single mouse from each group (*n* = 5) are shown. Color scales indicate relative luminescence (photon flux). The line graph shows average tumor bioluminescence signal over time, expressed as mean photon counts per second per cm^2^. **b**. Tumor volume by caliper measurement and tumor weights at the end of study on Day 8. Representative longitudinal whole-body PET images of tumor-bearing mice after i.v.i administration of the [^89^Zr]Zr-chDAB4 radioconjugate on Day 1. **c.** Relative extent of tissue uptake of activity is shown in the Maximum Intensity Projections of the whole body PET images according to the color scale. Arrows at Day 8 indicate tumors in right flank of each mouse. Tumor uptake of the [^89^Zr]Zr-chDAB4 radioconjugates during the course of the experiment was quantified and expressed as the percentage injected activity per gram (%IA/g) using the PMOD® software. **d**. Organs were removed on Day 8 and the biodistribution of [^89^Zr]Zr-chDAB4 was measured using a Hidex counter**.** P values were measured by two-way ANOVA. All data points are means ± SEM.

In the subcutaneous A2780 ovarian cancer xenograft model, cisplatin treatment significantly controlled tumor growth compared to untreated control mice as measured using bioluminescence imaging (Fig. 4a). The significant antitumor activity of cisplatin was also evident both as reduced tumor growth by caliper measurements and lower *ex vivo* tumor weights at the end of study (Fig. 4b), with the mean tumor weight of cisplatin-treated mice being 196 ± 82.1 mg compared to 563 ± 60.8 mg in untreated mice*.*

Mice were imaged daily after injection of [^89^Zr]Zr-chDAB4 on Day 1, the day after chemotherapy administration, and representative PET scans together with the extent of tumor uptake determined by PMOD PET image analysis are shown in Fig. 4c. These data show that tumor uptake of [^89^Zr]Zr-chDAB4 significantly increased in treated mice at 2–3 days post-[^89^Zr]Zr-chDAB4 injection, which corresponds to 3 and 4 days after chemotherapy (given on Day 0). In Fig. 4c, the mean tumor uptake as %IA/g ± SEM at 2–3 days post-[^89^Zr]Zr-chDAB4 injection was 49.3 ± 3.1 and 49.3 ± 3.7, respectively, for cisplatin-treated mice compared to 37.9 ± 3.9 and 37.6 ± 3.9, respectively, for untreated mice. Again, this tumor uptake of [^89^Zr]Zr-chDAB4 in cisplatin-treated mice preceded the later observed delay in tumor growth (Fig. 4a).

Organ assay at the completion of the study showed elevated, but not statistically significant, tumor accumulation in cisplatin-treated mice, with no significant differences in uptake in normal tissues (Fig. 4d).

**Fig. 5.**
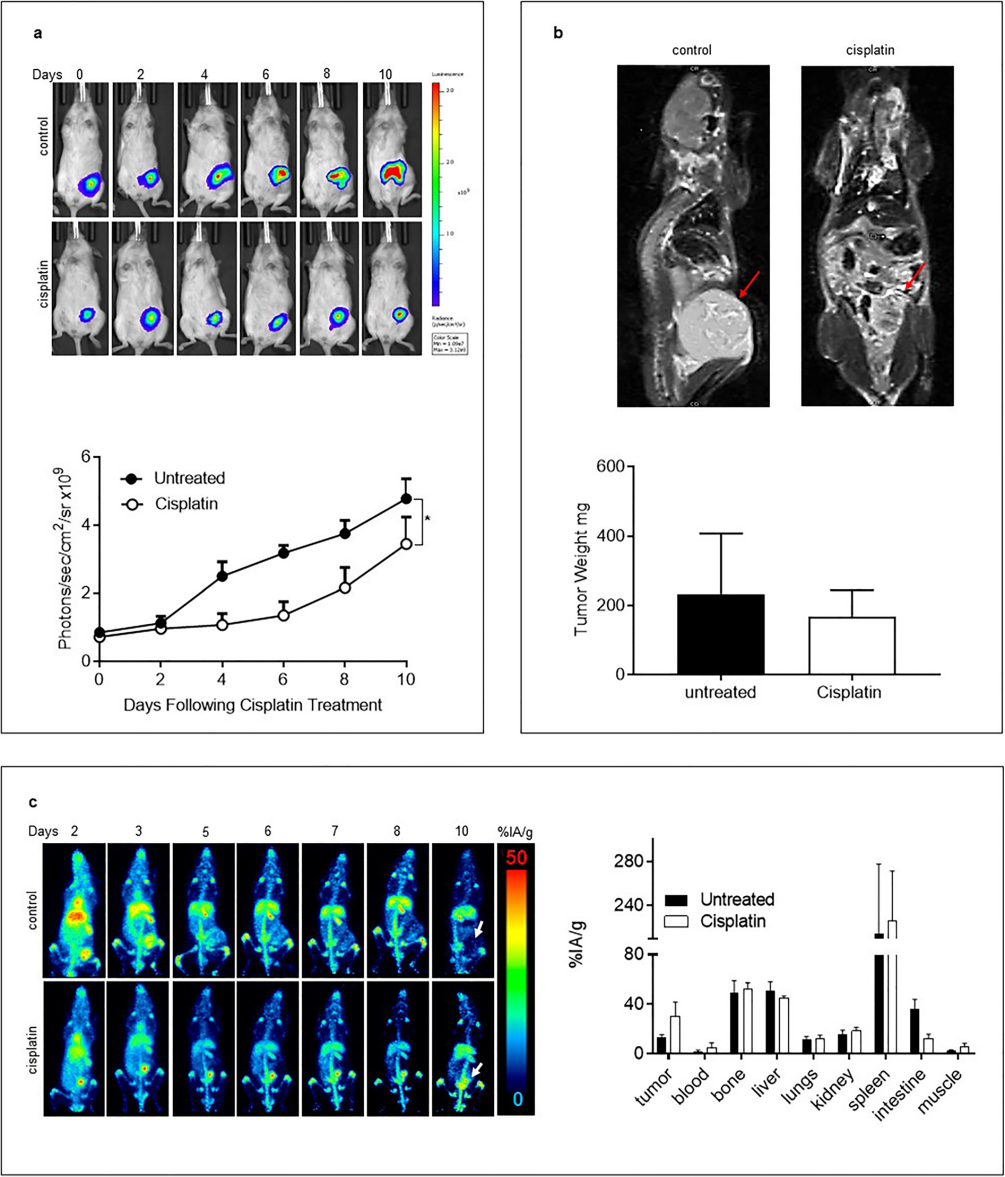
Biodistribution of [^89^Zr]Zr-chDAB4 in mice bearing ascitic tumors of A2780 human ovarian cancer. **a**. Representative longitudinal whole-body bioluminescence images of single mice from each group (*n* = 3) are shown (top panel). Color scales indicate relative luminescence (or photon flux). Line graph shows average tumor bioluminescence signal over time, expressed as mean photon counts per second per cm^2^(bottom panel). **b**. At the end of the study on Day 10, MRI scans taken of the same pair of mice show intraperitoneal tumors (arrows) and tumors were removed and weighed. **c**. Representative longitudinal Maximum Intensity Projections of whole-body PET images of a single mouse from each group showing the relative extent of tissue uptake of activity are shown. Arrows at Day 10 time point indicate tumors in right flank of each mouse. In the upper control panel, the arrow indicates a PET signal void, which corresponds to the tumor arrowed on the MRI scan. In the lower treatment panel, the arrow marks uptake by the shrunken peritoneal tumor. Organs were removed on Day 10 and the biodistribution of [^89^Zr]Zr-chDAB4 was measured using a Hidex counter. All data points are means ± SEM. P values were measured by two-way ANOVA.

A2780 tumor cells, which can also be grown as ascitic tumors, were given by intraperitoneal injection to NSG mice to reproduce the pattern of tumor growth commonly seen in ovarian cancer patients. Bioluminescence imaging was used to evaluate tumor growth because the ascitic tumors are not easily palpable. Representative whole-body bioluminescence images (Fig. 5a) and quantification of these data showed a significant reduction in disease burden compared to untreated mice, particularly within the first few days after chemotherapy. MRI imaging also confirmed the presence of an intraperitoneal tumor and reduced tumor size after cisplatin treatment. At the completion of the experiment, mean tumor weight (± SEM) in cisplatin-treated mice was smaller 168.8 mg (± 92.1) than in untreated mice 233.3 mg (± 173.9), but did not reach statistical significance (Fig. 5b). Representative PET slices and the biodistribution of [^89^Zr]Zr-chDAB4 (Fig. 5c) indicated that tumor accumulation of [^89^Zr]Zr-chDAB4 tended to be higher in cisplatin-treated mice, 30.3 ± 11.5 %IA/g, than in untreated mice, 13.3 ± 2.2 %IA/g, but did not reach statistical significance.

## Discussion

This study shows that radiolabeling chDAB4 with Zirconium-89 enables noninvasive and longitudinal study of its biodistribution in live mice bearing lung or ovarian cancer xenografts. In current clinical oncology practice, interim responses to platinum-based chemotherapy may not be determined by CT scanning until after 2 or 3 cycles of chemotherapy (equivalent to 9 or 12 weeks), which may mean that patients experience chemotherapy side effects without realizing a clinical benefit [45]. Our whole-body PET imaging studies in mice bearing cisplatin-responsive human ovarian and lung cancers [46, 47] provide preclinical proof of concept for [^89^Zr]Zr-labeled chDAB4 as a tumor-specific marker of postchemotherapy cell death, which indicates a tumor response to treatment earlier than that apparent later as tumor growth delay.

As shown in Fig. 1, cisplatin treatment induces dose-dependent binding of chDAB4 to dead PI^+^ tumor cells *in vitro*, which manifests both as an increasing proportion of chDAB4-bound dead cells as well as an increasing amount of chDAB4 that each dead tumor cell binds. This observation is similar to the time-dependent increases in both the number of PI^+^ cells bound by DAB4 and per cell binding of DAB4, which have been observed in previous *in vitro* and *in vivo* studies [27, 31]. Here, however, we expect that this cisplatin dose-dependent increase in tumor cell death represents an accelerating process of post-apoptotic necrosis of which chDAB4 binding provides a sensitive measure of both its extent and intensity. Although we do not fully understand this phenomenon, based on our previous studies we hypothesize that the cellular epitope of La/SSB becomes increasingly available *in vivo* for chDAB4 binding during the process of post-apoptotic necrosis [30].

The postchemotherapy patterns of tumor uptake observed with [^89^Zr]Zr-labeled chDAB4 in the animal biodistribution studies are consistent with results of previous biodistribution studies in murine tumor models using ^14^C-, ^111^In-, ^90^Y-, ^177^Lu- or ^227^Th-labeled versions of the parental murine DAB4 mAb [27, 31]. In these studies, postchemotherapy tumor accumulation of DAB4 was rapid, antigen-specific and associated with the extent of chemotherapy-induced tumor cell death. Notwithstanding the effect of the prior step of DNA-damaging chemotherapy on tumor uptake of DAB4, there was no evident accumulation of DAB4 in chemo-sensitive normal tissues such as intestine.

The observed uptake of chDAB4 within tumors of untreated mice results from the presence of spontaneously apoptotic and necrotic tumor cells, which are often located within the necrotic tumor core and which are a common pathophysiologic feature of solid tumors. It is likely that many of these necrotic tumor cells result from the death of hypoxic tumor cells as we have shown previously [31]. Using the highly chemosensitive syngeneic murine EL4 lymphoma model where chemotherapy is known to induce tumor cell apoptosis, we showed that the greatest and most intense uptake of DAB4 occurred in tumor cells dying as a result of chemotherapy rather than as a result of spontaneous necrosis [15]. In the current study, however, we selected the human H460 and A2780 tumor xenografts to better represent relatively chemoresistant human lung and ovarian carcinomas, respectively.

Our investigation of antibody drug conjugates of chDAB4 demonstrated that chDAB4-bound dead lung cancer cells could be phagocytosed *in vitro* by macrophages and suggested that similar macrophage-mediated processing of chDAB4 may occur *in vivo* [16, 17]. The reduced bone uptake of [^89^Zr]Zr^IV^ after prior blockade with unlabeled chDAB4 indicates that less free bone-seeking [^89^Zr]Zr^IV^ [48] is generated within the body of each mouse and this result may thus help to explain the higher blood activity level. These data suggest that catabolism of [^89^Zr]Zr-labeled chDAB4 is required to release free or chelator-bound [^89^Zr]Zr^IV^ and we hypothesize that tumor-associated macrophages (TAMs) are likely to be one of the major catabolizing cell types. In our recent study, we found some support for this hypothesis.

Tumor-bearing mice, which had been depleted of macrophages, were observed to have significantly less tumor accumulation of [^89^Zr]Zr-chDAB4 [17]. Zirconium-89 is a residualizing radionuclide, meaning that after internalization of the cell surface molecule targeted by a [^89^Zr]Zr-labeled mAb, the radiometal with or without its chelator is trapped inside the cell [49]. In the case of [^89^Zr]Zr-labeled chDAB4, our data indicate that TAMs are responsible for tumor residualization of [^89^Zr]Zr^IV^ [17].

After administration of [^89^Zr]Zr-labeled chDAB4 equivalent to a protein mass of between 50 μg and 75 μg, we observed bone uptake of [^89^Zr]Zr^IV^ in each of the three tumor models irrespective of the use of chemotherapy. Others have shown that administration of as little as 0.31 MBq/5μg [^89^Zr]Zr-labeled DS-8273a, which is a death receptor 5 targeting antibody, results in double the bone uptake of radionuclide compared to ^111^In-labeled DS-8273a [50]. Moreover, bone uptake of [^89^Zr]Zr^IV^ after administration of [^89^Zr]Zr-labeled mAbs can confound the interpretation of immunoPET images by potentially creating false positive osseous lesions [51]. In addition, in all three xenograft models, we observed significant splenic accumulation of radiolabel regardless of prior chemotherapy, which has been shown in other studies of immuno-incompetent NSG mice that had received radiolabeled antibodies [52–54].

The commercially available DFO-*p*Phe-NCS bifunctional chelator used in this study may have reduced *in vivo* stability [55, 56], thus contributing to bone accumulation of activity. Hence, the stability of the chelator together with the antibody concentration and labeled radioactivity remain important considerations in planning clinical applications of [^89^Zr]Zr-labeled mAbs [57]. In this respect, new chelators are being developed that may improve the stability and biodistribution of [^89^Zr]Zr-labeled mAbs such as [^89^Zr]Zr-chDAB4, which can generate free [^89^Zr]Zr^IV^, resulting in its uptake by bone. For example, Donnelly *et al.* compared their novel chelator, DFO-Sq, which is a squaramide ester derivative of DFO-*p*Phe-NCS, to DFO-*p*Phe-NCS using the HER2-targeting antibody, trastuzumab, and showed improved radiolabeling efficiency and PET imaging characteristics resulting from use of the DFO-Sq chelator [58]. Similarly, in a study of [^89^Zr]Zr-chDAB4, we found that DFO-Sq as a chelator led to better *in vitro* and *in vivo* stability and PET imaging quality than DFO-*p*Phe-NCS [41]. Another recent study confirmed the very good shelf stability and high chemical purity of a [^89^Zr]Zr-labeled immunoconjugate employing the DFO-Sq chelator [59].

In summary, we used [^89^Zr]Zr-chDAB4 immunoPET in mice bearing tumor xenografts of lung and ovarian cancer to show a specific, significant and early increase in postchemotherapy tumor binding of this necrotic radioligand, which pre-empted later tumor growth delay. The results of the *in vitro* CDC, ADCC, and cytokine release assays [60] demonstrate that the chimeric version of DAB4, which contains the K322A mutation in the CH2 domain of its huIgG1 Fc [38], lacks significant immune effector activity that might compromise its safety as a clinical radiodiagnostic agent. Therefore, the favorable immunotargeting and immunological profile of [^89^Zr]Zr-chDAB4 demonstrated in this study together with improved PET imaging and stability characteristics exhibited by the [^89^Zr]Zr-labeled DFO-Sq conjugate of chDAB4 [57] culminated in our decision to use the [^89^Zr]Zr-labeled DFO-Sq conjugate of chDAB4 in our currently open phase 1 immunoPET/CT trial ( ANZCTR No. 12620000622909). Although this clinical study is in progress, we show preliminary PET/CT data in two metastatic lung cancer patients who were not having concurrent anticancer treatment but who had FDG-avid metabolically active metastases (Supplementary Figure 2). Although these two patients lacked tumor uptake of [^89^Zr]Zr-DFO-Sq-chDAB4, as we have shown preclinically [14, 15], we hypothesize that this radiodiagnostic agent is sufficient to discern those malignant lesions that are responding to treatment by their extent of tumor uptake of [^89^Zr]Zr-DFO-Sq-chDAB4.

Of potential clinical utility, [^89^Zr]Zr-labeled chDAB4 can be used in radiation dosimetry [61] in order to determine subsequent dosing regimens for chDAB4 to be employed in clinical antibody radioconjugate therapy. Now that platinum-based chemotherapy is a component of standard frontline chemo-immunotherapy regimens for advanced NSCLC patients [62, 63], tumor-targeted internal radiotherapy such as that delivered by chDAB4 may lead to interactions with immune checkpoint inhibitor therapy and potentially promote the antitumor activity of the combination regimen [64].

In conclusion, although we provide preclinical proof-of-concept for [^89^Zr]Zr-chDAB4 immunoPET as a radiodiagnostic method to enable early detection of interim tumor responses to the platinum-based chemotherapy, we recognize that our results show that the degree of chDAB4 uptake by tumors of untreated mice may limit interpretation of treatment effects and thus the potential clinical utility of our technique. In our current phase 1 clinical immunoPET trial, only a single postchemotherapy dose of [^89^Zr]Zr-DFO-Sq-chDAB4 will be administered because as a chimeric antibody, chDAB4 may elicit untoward immune reactions. However, in future clinical studies we will mitigate this risk by using a humanized form of DAB4 so that we can also address the question of relative postchemotherapy tumor uptake of the radioligand by comparing its biodistribution between pre- and post-chemotherapy injections.

## Supplementary Information

ESM 1 Conjugated chDAB4 lacks immune effector functions. Fresh PBMCs from 5 normal volunteer donors were incubated for 24 h with increasing doses of chDAB4 or 15 μg/mL anti-CD3 (clone OKT3) as a positive control or 15 μg/mL anti-EGFR chimeric mAb cetuximab (Cetux.), as a negative control. Supernatant was collected and cytokine production analyzed using Biolegend LEGENDplex Human Inflammation Panel 1. All data points are means ± SEM. (JPG 111 kb)

ESM 2 Maximum intensity projections of [^18^F]-FDG PET scans (left hand panels) and PET scans 4 h after injection with [^89^Zr]Zr-DFO-Sq-chDAB4 (right hand panels) for two different patients. Red arrows show locations of tumors. (JPG 55 kb)
