# Supplementary figures and images for: Positron Emission Tomographic Imaging of Tumor Cell Death Using Zirconium-89-Labeled APOMAB® Following Cisplatin Chemotherapy in Lung and Ovarian Cancer Xenograft Models
Source: Mol Imaging Biol. 2021 Jul 6;23(6):914–28. doi: 10.1007/s11307-021-01620-1 (PMC8578059; doi:10.1007/s11307-021-01620-1)

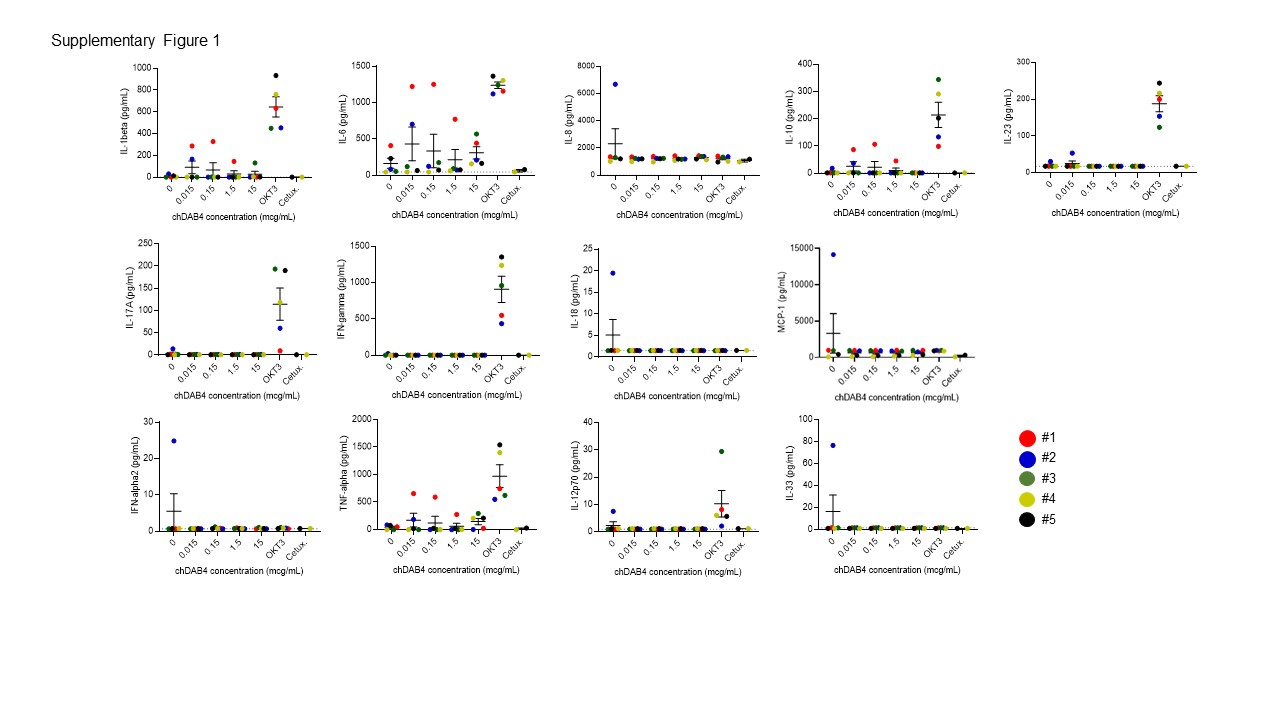

Supplement: Supplementary file 1 — Conjugated chDAB4 lacks immune effector functions. Fresh PBMCs from 5 normal volunteer donors were incubated for 24 h with increasing doses of chDAB4 or 15 μg/mL anti-CD3 (clone OKT3) as a positive control or 15 μg/mL anti-EGFR chimeric mAb cetuximab (Cetux.), as a negative control. Supernatant was collected and cytokine production analyzed using Biolegend LEGENDplex Human Inflammation Panel 1. All data points are means ± SEM. (JPG 111 kb) [file 11307_2021_1620_MOESM1_ESM.jpg]

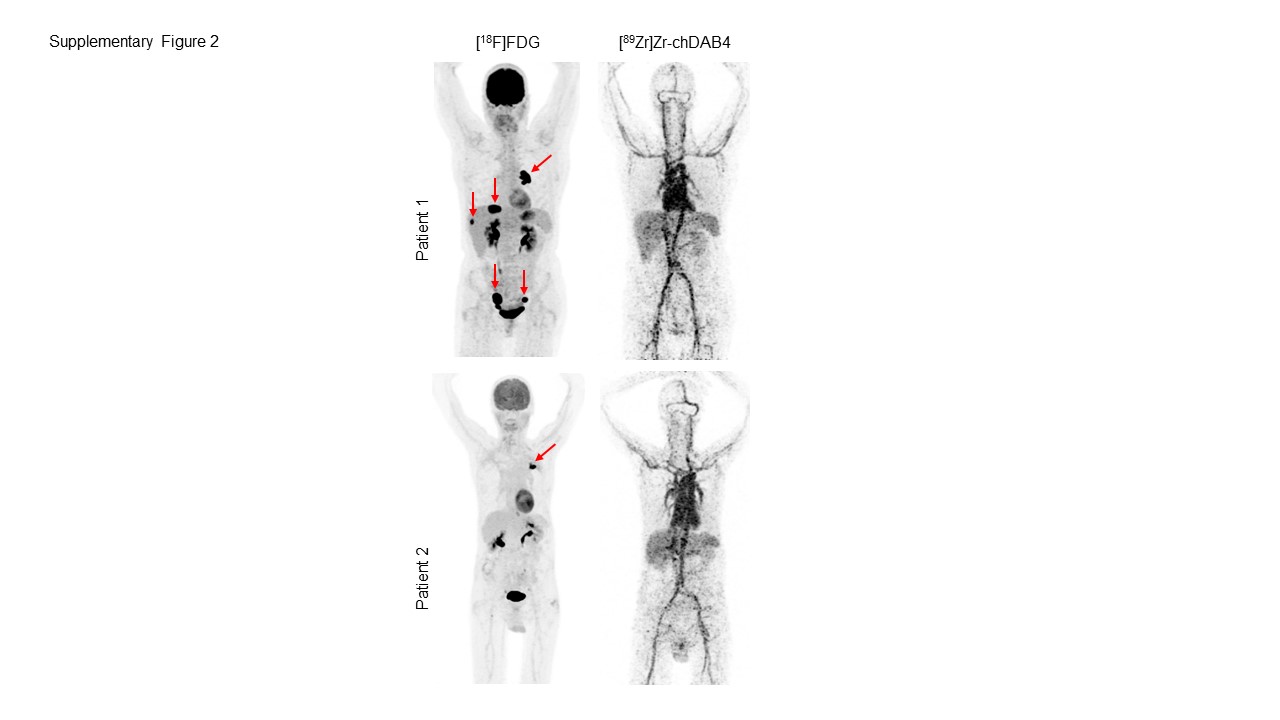

Supplement: Supplementary file 2 — Maximum intensity projections of [18F]-FDG PET scans (left hand panels) and PET scans 4 h after injection with [89Zr]Zr-DFO-Sq-chDAB4 (right hand panels) for two different patients. Red arrows show locations of tumors. (JPG 55 kb) [file 11307_2021_1620_MOESM2_ESM.jpg]
